# A novel *CHD7* variant disrupting acceptor splice site in a patient with mild features of CHARGE syndrome: a case report

**DOI:** 10.1186/s12881-019-0859-y

**Published:** 2019-07-17

**Authors:** Evelina Siavrienė, Gunda Petraitytė, Violeta Mikštienė, Tautvydas Rančelis, Živilė Maldžienė, Aušra Morkūnienė, Jekaterina Byčkova, Algirdas Utkus, Vaidutis Kučinskas, Eglė Preikšaitienė

**Affiliations:** 1 Department of Human and Medical Genetics, Faculty of Medicine, Institute of Biomedical Sciences, Vilnius University, Vilnius, Lithuania; 2 Center of Ear, Nose and Throat Diseases, Vilnius University Hospital Santaros Clinics, Vilnius, Lithuania

**Keywords:** *CHD7*, C.5535-1G > A splice site variant, CHARGE syndrome, Congenital anomalies, cDNA analysis

## Abstract

**Background:**

CHARGE syndrome (MIM# 214800)—which is characterised by a number of congenital anomalies including coloboma, ear anomalies, deafness, facial anomalies, heart defects, atresia choanae, genital hypoplasia, growth retardation, and developmental delay—is caused by a heterozygous variant in the *CHD7* (MIM# 608892) gene located on chromosome 8q12. We report the identification of a novel c.5535-1G > A variant in *CHD7* and provide the evaluation of its effect on pre-mRNA splicing.

**Case presentation:**

In this study, we report on a female presenting features of CHARGE syndrome. A novel heterozygous *CHD7* variant c.5535-1G > A located in the acceptor splice site of intron 26 was identified in the proband’s DNA sample after analysis of whole exome sequencing data. In silico predictions indicating that the variant is probably pathogenic by affecting pre-mRNA splicing were verified by genetic analysis based on reverse transcription of the patient’s RNA followed by PCR amplifications performed on synthesised cDNA and Sanger sequencing. Sanger sequencing of cDNA revealed that the c.5535-1G > A variant disrupts the original acceptor splice site and activates a cryptic splice site only one nucleotide downstream of the pathogenic variant site. This change causes the omission of the first nucleotide of exon 27, leading to a frameshift in the mRNA of the *CHD7* gene. Our results suggest that the alteration induces the premature truncation of the CHD7 protein (UniProtKB: Q9P2D1), thus resulting in CHARGE syndrome.

**Conclusion:**

Genetic analysis of novel splice site variant underlines its importance for studying the pathogenic splicing mechanism as well as for confirming a diagnosis.

**Electronic supplementary material:**

The online version of this article (10.1186/s12881-019-0859-y) contains supplementary material, which is available to authorized users.

## Background

Rapid advancement of high-throughput next generation sequencing (NGS) technologies has provided the necessary conditions for the identification of disease-causing variants in the human genome [1]. Even though the technique for detecting variants is now becoming routine, the challenge of performing a detailed analysis and determining the function of the altered gene is still open [2].

According to data from the 1000 Genomes Project, a typical human genome contains 149–182 sites with protein truncating variants, more than 10,000 sites with peptide sequence altering variants, and about 459,000–565,000 variant sites affecting known regulatory regions [3]. Previous studies have shown that many genetic diseases have been caused by coding and non-coding variants disrupting splice sites [4, 5]. Variants altering pre-mRNA splicing account for at least 10% of disease-causing gene alterations [6].

The *CHD7* (Chromodomain Helicase DNA Binding Protein 7; MIM #608892; NM_017780.4) gene, which is located on chromosome 8q12, encodes protein that contains several helicase family domains [7]. Heterozygous variants within this gene are the major cause of CHARGE syndrome (MIM #214800; ORPHA: 138). This syndrome is a rare autosomal dominant disorder estimated to occur in about 1:10,000 births worldwide [8]. CHARGE syndrome is characterised by a number of different congenital anomalies, including Coloboma, Heart defects, Atresia of the choanae, Retardation of growth and development, Genital hypoplasia, Ear anomalies, and deafness [9, 10]. CHARGE syndrome shares several major findings and phenotypic overlap with Mandibulofacial dysostosis, Guion-Almeida type (MFDGA; MIM #610536; ORPHA: 79113) [11, 12] and Kabuki syndrome 1 (KABUK1; MIM #147920; ORPHA: 2322) [13], which are caused by heterozygous variants in *EFTUD2* (MIM #603892) and *KMT2D* (MIM #602113), respectively. There is some evidence that the phenotype can be caused by a variant in the *SEMA3E* (Semaphorin-3E; MIM #608166) gene [14], but almost 60–70% of patients have a pathogenic variant in the *CHD7* gene [15, 16]. In the CHD7 database [17, 18], 801 pathogenic variants in *CHD7* are currently recorded. According to the Human Gene Mutation Database (HGMD) [19, 20], the most frequent variants in the *CHD7* gene are nonsense, small deletions, and insertions. Moreover, a number of different pathogenic variants in *CHD7* splice sites have also been recorded.

This study aims to reveal the consequences and the pathogenicity of a novel heterozygous splice site c.5535-1G > A variant in the *CHD7* gene in a female patient with mild features of CHARGE syndrome.

## Case presentation

### Clinical findings

**Fig. 1:**
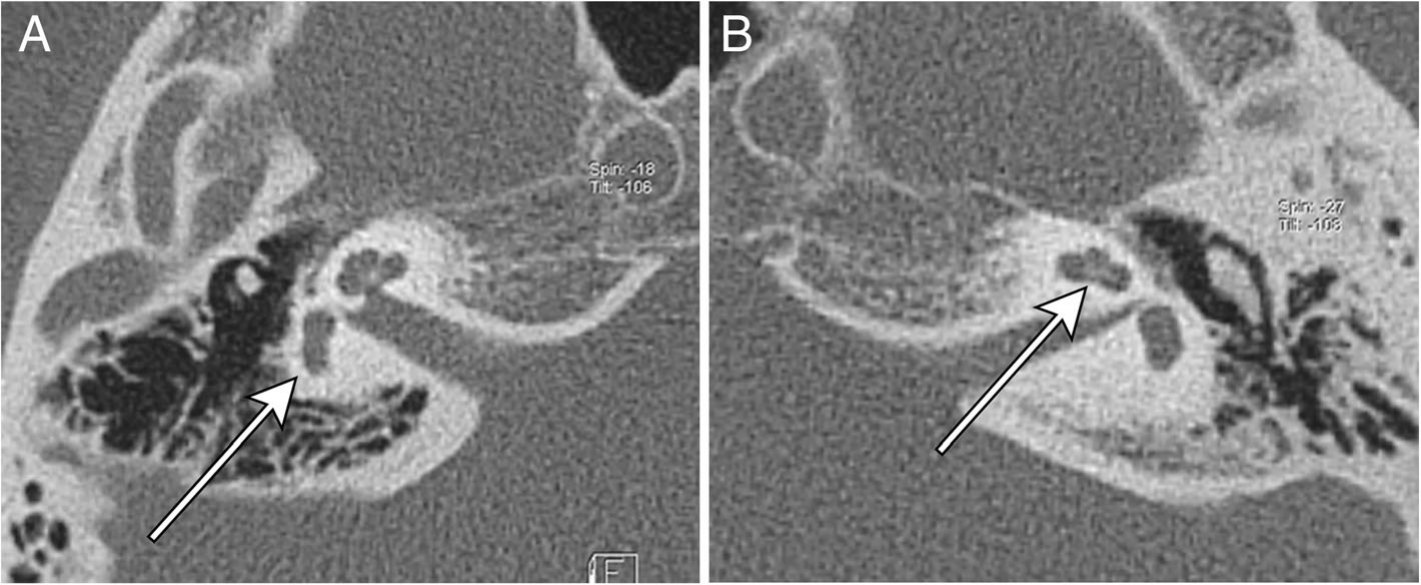
Temporal bone CT image showed aplasia of the semi-circular canals (**a**) and cochlear nerve canal atresia (**b**)

A female patient was referred for genetic assessment at 16 years of age for hearing impairment, scoliosis, and left side facial palsy. The proband was the second child of healthy unrelated Lithuanian parents aged 29 at the proband’s birth. The proband had neither a history of prenatal exposure to teratogenic agents nor any family history of congenital malformations. During the pregnancy, polyhydramnios was diagnosed. Congenital heart defect of the foetus was detected at 35 weeks of gestation. The proband was born at 38 weeks by Caesarian section due to congenital heart defect of the foetus. At birth, her weight was 2800 g (3rd centile), her length was 49 cm (10th centile), the circumference of her head was 35 cm (50th centile), and her Apgar score was 6 at 1 min and 8 at 5 min. Complete atrioventricular communication with a small ventricular septal defect, a moderately sized atrial septal defect with an aneurysm in the *interatrial septum*, and insufficiency of the mitral valve were diagnosed. Additionally, left side choanal atresia, left side facial palsy, a short neck, dysplasia of the sacrum, and dysplasia of the hip joint were observed. As a neonate, the patient had feeding difficulties and received a nasogastric tube for 1 month. Poor weight gain and cardiac insufficiency manifested at the age of 7 months. Auditory Brainstem Response Audiometry was performed and unilateral deafness in the left was diagnosed during infancy. Bilateral aplasia of the semicircular canals and cochlear nerve canal atresia on the left were found on temporal bone CT scans (Fig. 1). At the age of 1 year, radical surgical correction of atrioventricular communication was performed. Pyelonephritis and grade IV vesicoureteral reflux were first diagnosed at 1 year of age, and recurrent episodes of pyelonephritis continued until the age of 7 years. At age of 4 years, surgical repair of choanal atresia on the left was performed and a tympanostomy tube was placed in the same side due to recurrent otitis media. The choanal atresia operation was performed again at the age of 5–6 years. Bone-anchored hearing aid (BAHA) surgery was performed at 7 years of age. Due to a skin reaction at the location of the implant, another operation to implant a new BAHA attract system was performed after the device had been used for 4 years. Adenoidectomy was performed at 11 years of age. The proband had hypermetropia, for which glasses were prescribed. Scoliosis of 17° was evaluated at the age of 16 years.

Motor development of the proband was delayed; she walked alone at the age of 27 months. According to the DISC scale, at the age of 29 months her psychomotor development was evaluated at the range of 21 (gross motor) to 29 (fine motor, visual attention and memory) months. Eruption of permanent teeth was also mildly delayed. The girl attended a regular school and had no behavioural problems. Her psychological evaluation at the age of 16 years yielded a verbal score of 90, a performance score of 97, and a full score of 94 on the Wechsler Intelligence Scale for Children (WISC-III).

During an examination at the age of 16 years, her height was 162 cm (25th centile), her weight was 48.5 kg (3rd–10th centile), and the circumference of her head was 53.5 cm (3rd–10th centile). Her phenotype was remarkable for left side facial palsy, low forehead, deformed nasal bridge, synophrys, protruding ears, auricular pits, low posterior hairline, short neck, limited movements of the neck, pectus excavatum, hirsutism, and Tanner PH-4, B-3. Additionally, primary amenorrhoea and premature ovarian insufficiency were diagnosed at 18 years of age. She had low levels of estradiol and near normal concentrations of prolactin and follicle-stimulating hormone. A pelvic ultrasound visualised a uterus (49x15x31 mm) and ovaries (26-27 × 12 mm).

### Whole exome sequencing

Whole genomic DNA (gDNA) was extracted from peripheral blood of our proband and her healthy parents following the standard phenol-chloroform extraction protocol.

Whole exome sequencing (WES) using the high throughput next generation sequencing (NGS) technique was used to sequence the sample of the proband (Illumina, Inc., USA). DNA libraries generated using TruSeq Rapid Exome Library Prep kit (8x3plex) (Illumina, Inc., USA). In order to precisely measure the concentration of DNA libraries, Qubit dsDNA BR Assay kit (ThermoFisher Scientific, USA) and Qubit fluorimeter (ThermoFisher Scientific, USA) were used. Clusters amplificated using cBot system (Illumina, Inc., USA), TruSeq PE Cluster Kit v3-HS (Illumina, Inc., USA) and TruSeq Dual Index Sequencing Primer Box – Paired End (Illumina, Inc., USA). WES was performed using TruSeq SBS Kit v3-HS (Illumina, Inc., USA) and by employing the HiScanSQ (Illumina, Inc., USA) genetic analyzer.

Analyses of high throughput sequencing data started with alignment against The Human NCBI Build GRCh37 (hg19/2009) reference genome. The annotation of NGS data was made using the ANNOVAR v.2018Apr16 program. The pathogenicity of variants was assessed using ACMG criteria, taking into account the data provided by the ANNOVAR program, the available databases (ExAC Browser, 1000 Genome Project, NCBI dbSNP, NCBI dbVar, HGMD, NCBI OMIM, NCBI ClinVAR, Leiden Open Variation Database, NCBI Genome, Deafness Variation Database), and the relevant scientific literature. The pathogenic or probably pathogenic sequence variants were checked by analyzing proband’s BAM files using the visualization tool Integrative Genomics Viewer (IGV).

### Reverse transcription reaction

Total RNA of the proband and her healthy parents’ samples was extracted from whole blood using a Tempus™ Blood RNA- Tube and Tempus™ Spin RNA Isolation Kit (Thermo Fisher Scientific, USA) according to the optimized manufacturer’s protocols. Total RNA concentration and quality were checked using a NanoDrop 2000 spectrophotometer (Thermo Fisher Scientific, USA). Complementary DNA (cDNA) was reverse-transcribed (RT) from a total RNA using a High-Capacity RNA-to-cDNA Kit (Thermo Fisher Scientific, USA) and a ProFlex PCR system (Thermo Fisher Scientific, USA) following manufacturer’s protocol and recommended conditions.

### Polymerase chain reactions and Sanger sequencing

Polymerase chain reactions (PCR) of gDNA and cDNA sequences flanking splice site c.5535-1G > A variant of *CHD7* were performed using specific primers designed with Primer Blast tool [21, 22]. gDNA was PCR amplified using primer pair designed on exon 26 (forward primer) and on intron 27 (reverse primer), while PCR amplifications of cDNA sequence were performed using the same forward primer, but different reverse primers, which were designed on the 28–29 exon junction as well as on the 29–30 exon junction (Additional file 1: Table S1). PCR products were fractioned according to standard agarose gel electrophoresis.

gDNA samples was used for the segregation analysis. In order to elucidate the pathogenicity of detected variant, the cDNA samples were analysed. The PCR products were sequenced with BigDye® Terminator v3.1 Cycle Sequencing Kit (Thermo Fisher Scientific, USA) and ABI 3130xL Genetic Analyser (Thermo Fisher Scientific, USA). The sequences were aligned with the reference sequence of the *CHD7* (NCBI: NM_017780.4) gene.

### In silico analysis

Mutation Taster [23, 24] and Human Splicing Finder [25] databases were used for predicting splice site alterations. Sequences of evolutionary distinct species were obtained from the Ensembl genome browser [26, 27], while a sequence alignment was produced using Clustal Omega tool [28]. Possible splice site c.5535-1G > A variant’s effect on the CHD7 protein (UniProtKB: Q9P2D1) was predicted using different tools and databases, such as ExPASy Bioinformatics Resource Portal [29, 30] and Pfam 32.0 database [31, 32].

### Genetic findings

Heterozygous *CHD7* variant NC_000008.11(NM_017780.4):c.5535-1G > A, located in the acceptor splice site of intron 26, was identified in the proband’s gDNA sample after analysis of WES data.

The acceptor splice site variant is novel and not recorded in the CHD7 database, HGMD, or other databases. The variant was confirmed by Sanger sequencing on the proband’s gDNA sample. Segregation analysis by Sanger sequencing on the gDNA samples of her parents confirmed the de novo origin of the variant. The splice site variant was evaluated by in silico analysis.

**Fig. 2:**
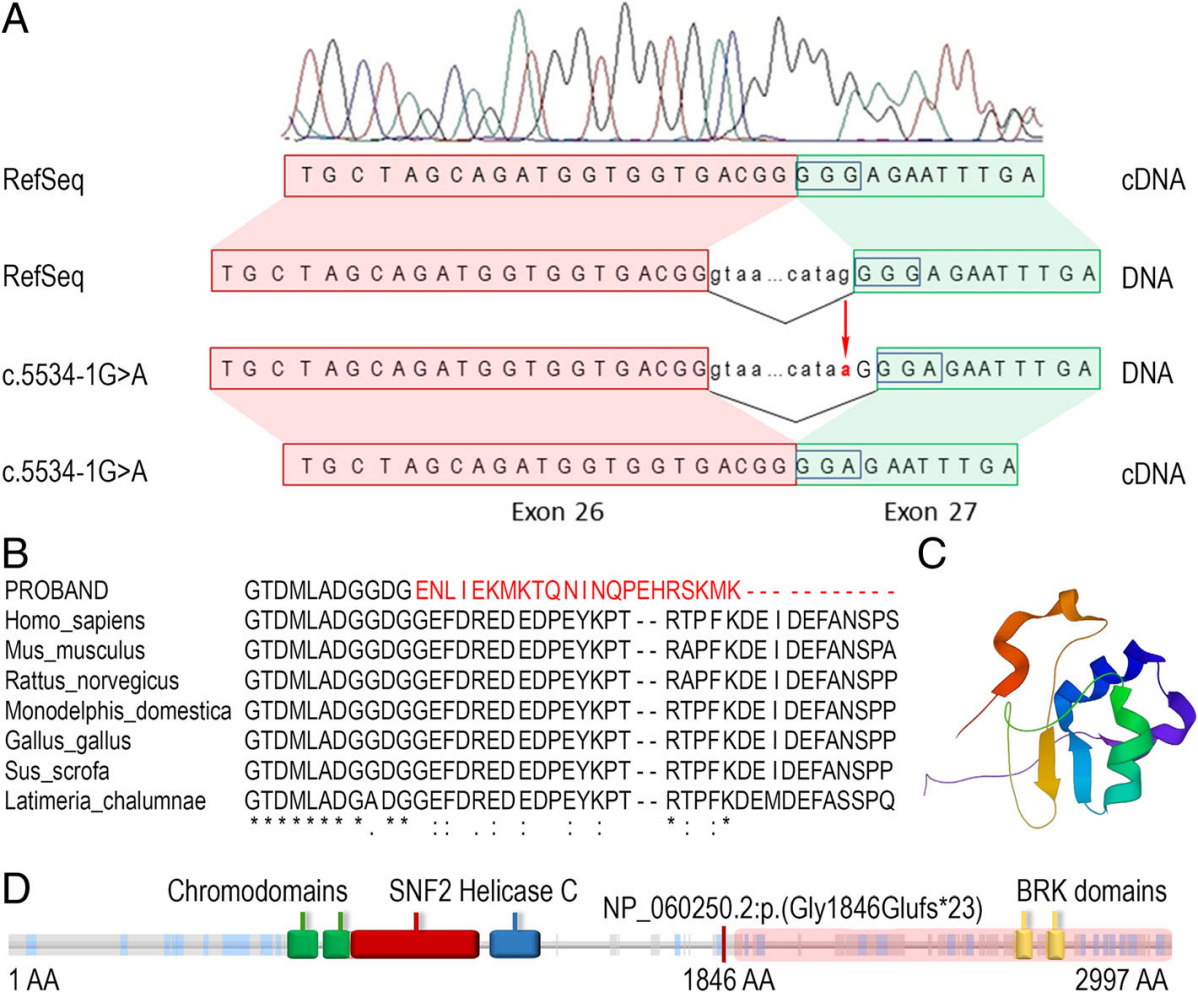
**a** The chromatogram and schematic representation of wild-type and mutated sequences illustrate the disrupted acceptor splice site at position − 1 of intron 26, which lead to a frameshift in the cDNA sample. **b** A comparative sequence alignment produced by ClustalO of the CHD7 protein across seven evolutionarily distant species. Frameshift of 23 new amino acids and therefore a truncated 1869 amino acid sequence is highlighted in red. Below the protein sequences is a key denoting conserved sequence (*), conservative variants (:), semi-conservative variants (.), and non-conservative variants (). **c** 3D model of the BRK domain, which is absent in the patient. Picture produced by the LiteMol program [33]. **d** A schematic representation of the CHD7 protein, which is 2,997 amino acids in length, with the arrangement of the main domains: two chromodomains (green), SNF2 domain (red), helicase domain (blue), and two BRK domains (yellow). A schematic view was adopted from the Pfam protein domain database

A variant effect prediction method, Mutation Taster, predicted the variant to be likely pathogenic and disease causing. Moreover, the alteration of the acceptor splice site was predicted by the Human Splicing Finder database. The variant was predicted to affect pre-mRNA splicing by the altering acceptor splice site at − 1 position and the possible use of a cryptic splice site. Sequence alignment of the CHD7 protein in seven species revealed that the region containing the splice site variant is highly conserved (Fig. 2b).

To study the potential pathogenicity of the *CHD7* gene c.5535-1G > A variant, the analysis of the cDNA sequence of the patient and healthy controls was performed. Sanger sequencing of the patient’s cDNA sample revealed that the c.5535-1G > A variant causes the loss of an original acceptor splice site at position − 1 in the intron 26 of *CHD7* and consequently activates a cryptic splice site only one nucleotide downstream of the pathogenic variant site (Fig. 2a). Meanwhile, Sanger sequencing of cDNA samples from healthy controls did not expose any alteration in the splicing mechanism. In silico, this variant leads to the frameshift of 23 new amino acids and therefore a truncated 1869 amino acid protein NP_060250.2:p.(Gly1846Glufs*23) (Fig. 2d). The truncated CHD7 protein contains two functionally important chromodomains, the SNF2 domain and helicase domain, but there is a loss of two BRK domains (Fig. 2c).

## Discussion and conclusions

Variants that affect pre-mRNA splicing and eventually translated proteins have been shown to be one of the most frequent causes of hereditary disorders [4]. The characterisation of splice site variants of unknown consequence and clinical significance is critical for appropriate and comprehensive genetic testing [34]. The most common consequence of splicing variants is skipping of one or more exons, but in certain cases the activation of cryptic 5′ (donor) or 3′ (acceptor) splice site and partial or full retention of specific introns may occur [35]. However, the effects of these variants on splicing are often not pursued as a cause of disease. Since there are different consequences of these variants, they are especially difficult to predict without mRNA or cDNA analysis [5].

In this study, we presented the characterisation of a previously unreported de novo heterozygous acceptor splice site c.5535-1G > A variant in the *CHD7* gene. Earlier studies of variants in the 3′ splice site have shown that transitions, as well as in this case guanine (G) substitution for adenine (A), occur much more frequently than transversions, and the most frequently mutated base of an aberrant 3′ splice site is G [36]. The patient carrying this acceptor splice site variant fulfils major diagnostic characteristics of CHARGE syndrome outlined by Blanke et al. [37] and updated by Verloes [38]: the proband has three major characteristics (unilateral choanal atresia, facial palsy, temporal bone abnormalities) and three minor characteristics (delayed puberty, delayed mylestones, cardiovascular malformation). The haploinsufficiency of the *CHD7* gene is assumed to be the pathogenic mechanism of CHARGE syndrome [10]. This mechanism is supported by studies in which a heterozygous knock-out mouse model was engineered. In these studies, heterozygous *Chd7* mice presented features similar to those associated with CHARGE syndrome, providing strong support to the importance of the hemizygosity of *CHD7* in this syndrome [39–41]. Heterozygous variants of the *CHD7* gene play important roles in a number of vitally important processes such as embryonic development, cell cycle arrest, apoptosis, transcriptional regulation, DNA repair, replication, and recombination. Furthermore, the CHD7 protein is essential for regulation of distal genomic elements that control the expression of critical neural crest transcriptional factors. For this reason, it is hypothesised that the loss of 50% of the CHD7 protein is insufficient to maintain normal function of this gene, thus resulting in clinical manifestation [10, 16, 42].

Our further investigation of the acceptor splice site c.5535-1G > A variant detected by WES aimed to elucidate the pathogenicity of the novel variant in the *CHD7* gene. In silico analysis predicted this variant to be likely pathogenic. Moreover, the sequence alignment of the CHD7 protein across seven evolutionarily distant species revealed that the region containing the variant at the acceptor splice site is highly conserved, thus suggesting that this region is fundamentally important in vertebrates and alterations could be pathogenic (Fig. 2b). Our following research was based on cDNA analysis of the *CHD7* gene transcript. Unexpectedly, the data obtained on the patient’s cDNA showed that the c.5535-1G > A variant does not lead to adjacent exon skipping or intron retention; instead it activates a cryptic 3′ splice site only one nucleotide downstream from the pathogenic variant site. Eng et al. [43] presented a similar study of the acceptor splice site c.4437-1G > A variant in the *ATM* gene, which caused the same consequences to the cDNA sequence. In both cases, the first nucleotide of a particular exon is a G. The cryptic 3′ splice site has therefore been created using the mutated A of the intron and the G of the exon. These findings could suggest that if the original 3′ splice site is mutated the subunits of U2 spliceosomal snRNAs recognises the next AG site, which is located downstream of the original acceptor splice site. However, Moon et al. [44] reported two abnormal transcripts of the *HRPT2* gene, also known as the *CDC73* gene, resulting from the same c.238-1G > A variant of intron 2. The first transcript eliminated the whole exon 3, while the another lacked the first 23 nucleotides of exon 3 due to the use of a cryptic acceptor splice site in exon 3. For this reason, the exact mechanism for how one cryptic splice site is selected over another within the exon remains unclear. According to literature that we reviewed and the results of our experiment, the variant at − 1 position in the acceptor splice site and the first G nucleotide in the adjacent exon sequence is quite frequent [45, 46]. However, such variants usually disturb the reading frame by exon skipping [35] instead of the slippage of the splicing mechanism only 1 nt downstream as in our case. Moreover, the possible effect of altered splice sites is usually not based on mRNA or cDNA analysis and is predicted by computational algorithms [45, 47, 48] though the exact effect of the specific variant should be verified in functional studies [49].

Further in silico analysis showed that the activation of the cryptic 3′ splice site, including the first G nucleotide of exon 27, consequently disrupted the reading frame of the patient’s cDNA sequence, resulting in a truncated 1,869 amino acid protein NP_060250.2:p.(Gly1846Glufs*23). The original CHD7 protein (UniProtKB: Q9P2D1), which is 2,997 amino acids in length, has two N-terminal chromodomains and an SNF2 family domain that are involved in a variety of cellular processes but mainly affect the regulation of transcription. It also contains a C-terminal conserved helicase domain as well as two C-terminal BRK domains. The function of the BRK domain is still unknown (Fig. 2), but it is hypothesised that this domain may interact with chromatin components that are important and unique to higher eukaryotes [50, 51]. In our study, the truncated CHD7 protein is predicted to result in the loss of two BRK domains.

Based on these findings, our hypothesis is that the heterozygous acceptor splice site c.5535-1G > A variant in the *CHD7* gene results in partial haploinsufficiency of the *CHD7* gene in a patient with suspected CHARGE syndrome, because the variant causes premature termination of the translation of the mutated allele. The partial haploinsuficiency of the *CHD7* gene could also clarify the mild features of CHARGE syndrome that are characteristic of our patient. The results reported here explain the pathogenicity of this novel variant and predict the possible effect on the CHD7 protein, but further proteomic experiments are needed to clarify and validate the molecular effects caused by the c.5535-1G > A splice variant on the CHD7 protein.

In conclusion, despite a growing number of reported splicing variants and associated phenotypes, the effect on the genotype and the phenotype persists difficult to demonstrate, and therefore it remains one of the principal challenges in human molecular genetics. Our study showed that the novel splice acceptor variant c.5535-1G > A has a complete impact on the splicing of the *CHD7* gene, and the effect is predicted to be a truncated protein, thus resulting in CHARGE syndrome. These results enhance the importance of studying the pathogenicity of the splicing variant at the cDNA level and provide an opportunity to validate the suspected diagnosis and tailor clinical management for this family. Finally, the cDNA analysis provides a unique possibility to understand the etiology and pathophysiology and identify the molecular basis of many hereditary diseases and conditions, including those associated with congenital anomalies.

## Additional file

Additional file 1: **Table S1.** The conditions for PCR amplification of *CHD7* gene in gDNA and cDNA samples. (DOCX 55 kb)

## Data Availability

The main data generated and analysed during this study are included in this article and its supplementary information file. Any additional information is available from the authors upon request.

## Abbreviations

A: Adenine
*ATM*: Gene symbol for gene that encodes the protein ATM Serine/Threonine kinase
BAHA: Bone-anchored hearing aid
*CDC73*: Gene symbol for gene that encodes the protein cell division cycle 73
cDNA: Complementary DNA
*CHD7*: Gene symbol for gene that encodes the protein chromodomain helicase DNA binding
CT: Computed tomography
*EFTUD2*: Gene symbol for gene that encodes the protein elongation factor Tu GTP-binding domain-containing 2
G: Guanine
gDNA: Genomic DNA
HGMD: Human gene mutation database
*HRPT2*: Gene symbol for gene that encodes the protein hyperparathyroidism 2
IGV: Integrative genomics viewer
KABUK1: Kabuki syndrome 1
KID: Keratitis-ichthyosis-deafness syndrome
*KMT2D*: Gene symbol for gene that encodes the protein lysine-specific methyltransferase 2d
MFDGA: Mandibulofacial dysostosis, Guion-Almeida type
NGS: Next generation sequencing
PCR: Polymerase chain reaction
RefSeq: Reference sequence database in NCBI
RT: reverse transcription
*SEMA3E*: Gene symbol for gene that encodes the protein semaphorin-3E
WES: Whole exome sequencing
WISC-III: Wechsler intelligence scale for children
