# Supplementary material for: A novel CHD7 variant disrupting acceptor splice site in a patient with mild features of CHARGE syndrome: a case report
Source: BMC Med Genet. 2019 Jul 17;20:127. doi: 10.1186/s12881-019-0859-y (PMC6637606; doi:10.1186/s12881-019-0859-y)
Supplement: Supplementary file 1 — Table S1. The conditions for PCR amplification of CHD7 gene in gDNA and cDNA samples. (DOCX 55 kb) [file 12881_2019_859_MOESM1_ESM.docx]

**Supplementary Table 1.** The conditions for PCR amplification of *CHD7* gene in gDNA and cDNA samples.

| Gene  (RefSeq transcript) | Sample | Primers (5’ 🡪 3’) | Location | Annealing temperature | Amplicon length |
| --- | --- | --- | --- | --- | --- |
| *CHD7*  (NM_017780.4) | **gDNA**  for segregation analysis | Forward:  TCGGTATGCCTGATGCCAAG | Exon 26 | 64 ^0^C | 713 |
|  |  | Reverse: CCTTGAAAGCAAAGCAAGAAACAG | Intron 27 |  |  |
|  | **cDNA**  for functional analysis | Forward:  TCGGTATGCCTGATGCCAAG | Exon 26 | 65 ^0^C | 220 |
|  |  | Reverse: CTCTCACTGTGCTTGCCTGT | 28-29  exon junction |  |  |
|  | **cDNA**  for functional analysis | Forward:  TCGGTATGCCTGATGCCAAG | Exon 26 | 64 ^0^C | 444 |
|  |  | Reverse:  TTCTTGTCCACTTTTGCCGC | 29-30  exon junction |  |  |
